# Calcium Alginate Fibers/Boron Nitride Composite Lithium-Ion Battery Separators with Excellent Thermal Stability and Cycling Performance

**DOI:** 10.3390/molecules29225311

**Published:** 2024-11-11

**Authors:** Xing Tian, Hailing Shi, Linfeng Wang, Lupeng Shao, Liwen Tan

**Affiliations:** 1 State Key Laboratory of Biomaterials and Green Papermaking, Qilu University of Technology, Jinan 250306, China; shaolp@qlu.edu.cn; 2 State Key Laboratory of Bio-Fibers and Eco-Textiles, School of Material Science and Engineering, Institute of Marine Bio-Based Materials, Qingdao University, Qingdao 266071, China; 17864271858@163.com (H.S.); wlf2676630205@163.com (L.W.)

**Keywords:** separator, safe lithium-ion batteries, boron nitride, calcium alginate fiber

## Abstract

As one of the most critical components in lithium-ion batteries (LIBs), commercial polyolefin separators suffer from drawbacks such as poor thermal stability and the inability to inhibit the growth of dendrites, which seriously threaten the safety of LIBs. In this study, we prepared calcium alginate fiber/boron nitride-compliant separators (CA@BN) through paper-making technology and the surface coating method using calcium alginate fiber and boron nitride. The CA@BN had favorable electrolyte wettability, flame retardancy, and thermal dimensional stability of the biomass fiber separator. Meanwhile, the boron nitride coating provided excellent thermal conductivity and mechanical strength for the composite separator, which inhibited the growth of lithium dendrites and enabled lithium-ion symmetric batteries to achieve more than 1000 stable and long cycles at a current density of 0.5 mA cm^−2^. The interwoven fiber mesh formed by the boron nitride coating and the calcium alginate provided multiple pathways for ion migration, which enhanced the storage capacity of the electrolyte, improved the interfacial compatibility between the separator and the electrode, widened the window of electrochemical stability, and enhanced ionic migration. This eco-friendly bio-based separator paves a new insight for the design of heat-resistance separators as well as the safe running of LIBs.

## 1. Introduction

Lithium-ion batteries play an increasingly important role in modern society. As a critical component of lithium-ion batteries, the separator prevents electrode contact and provides a channel for ion transport. Conventional polyolefin separators tend to shrink at high temperatures, which increases the chance of electrode contact, resulting in short-circuit failures and even fires and explosions [1,2,3]. In addition, polyolefin separators are difficult to degrade, which is not conducive to the recycling of lithium batteries and leads to environmental pollution [4,5,6]. Therefore, the separator’s heat resistance and dimensional stability are essential to the safety of lithium-ion batteries.

Numerous studies have shown that surface coating methods can enhance the thermal and electrochemical stability of polyolefin separators effectively [7,8,9,10]. For example, Tan et al. [11] improved polyethylene separators’ thermal stability and lithiumphilicity by coating silicon oxide particles on their surface. Liu et al. [12] increased the cycling stability of the separators by coating hexagonal boron nitride on polypropylene. Sheng et al. [13] combined hexagonal boron nitride with polyimide to coat commercial polypropylene separators, which inhibited the growth of lithium dendrites and improved thermal stability. However, because of the limitation of polyolefin matrix materials, the above-mentioned compliant separators did not change the problem of the non-degradability of conventional separators. In recent years, biomass separators based on biopolysaccharide fibers (cellulose fibers and alginate fibers) have received widespread attention due to their multilayered porous structure and the large number of polar groups in the molecular chain, which exhibit excellent electrolyte wettability, flame retardancy, and thermal dimensional stability [14,15,16].

**Scheme 1:**
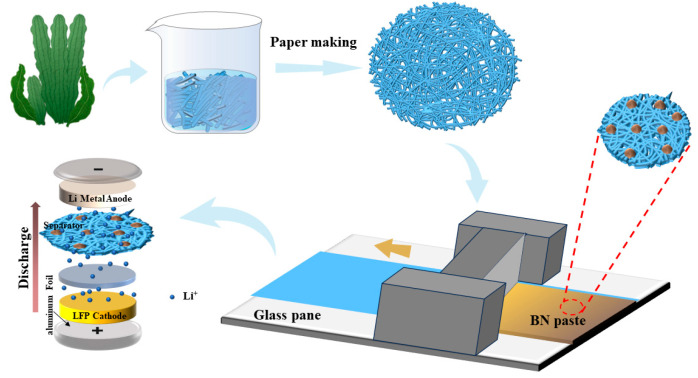
Preparation for CA@BN.

In this study, we prepared calcium alginate fiber/boron nitride-compliant separators (CA@BN) through paper-making technology and the surface coating method using calcium alginate fiber and boron nitride (Scheme 1). The CA@BN had favorable electrolyte wettability, flame retardancy, and thermal dimensional stability of the biomass fiber separator. Meanwhile, the boron nitride (BN) coating provided excellent thermal conductivity and mechanical strength for the composite separator, which inhibited the growth of lithium dendrites and enabled lithium-ion symmetric batteries to achieve more than 1000 stable and long cycles at a current density of 0.5 mA cm^−2^. The interwoven fiber mesh formed by the BN coating and the calcium alginate (CA) provided multiple pathways for ion migration, which enhanced the storage capacity of the electrolyte, improved the interfacial compatibility between the separator and the electrode, widened the window of electrochemical stability, and enhanced ionic migration.

## 2. Results and Discussion

### 2.1. Structure and Properties

**Figure 1:**
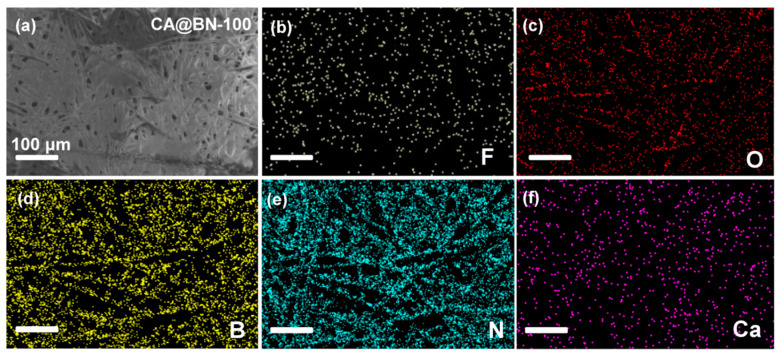
(**a**) Top-view SEM images for CA@BN-100 separators. EDS elemental mapping of (**b**) F, (**c**) O, (**d**) B, (**e**) N, and (**f**) Ca.

The morphology and coating thickness of the separator were evaluated using SEM and EDS. Figure 1a shows that CA@BN-100 demonstrated the formation of a compact flake of boron nitride powder on the CA separator, efficiently occupying its wide pores. Elemental mapping images of CA@BN-100, shown in Figure 1b–f, show the presence of elements F, O, B, N, and Ca. Table S1 shows that the separator created with a 100 μm applicator had significantly higher levels of boron (B) and nitrogen (N), with percentages of 32.2% and 46.08% correspondingly. The results indicated that BN did not completely block the pores of the fibers during the fabrication of the CA@BN separator due to the complex and intricate structure of the CA separator, which consists of multiple layers of fibers that are randomly intertwined and have pores of different sizes. Instead, it effectively reduced the size of the pores. In addition, we obtained samples with three different coating thicknesses, labeled CA@BN-50, CA@BN-100, and CA@BN-200, on a 73 μm CA separator by varying the coating thickness. As shown in Figure S1, the total thickness of the separator rose to 88 μm, 110 μm, and 129 μm with the coating thickness increase, respectively.

**Figure 2:**
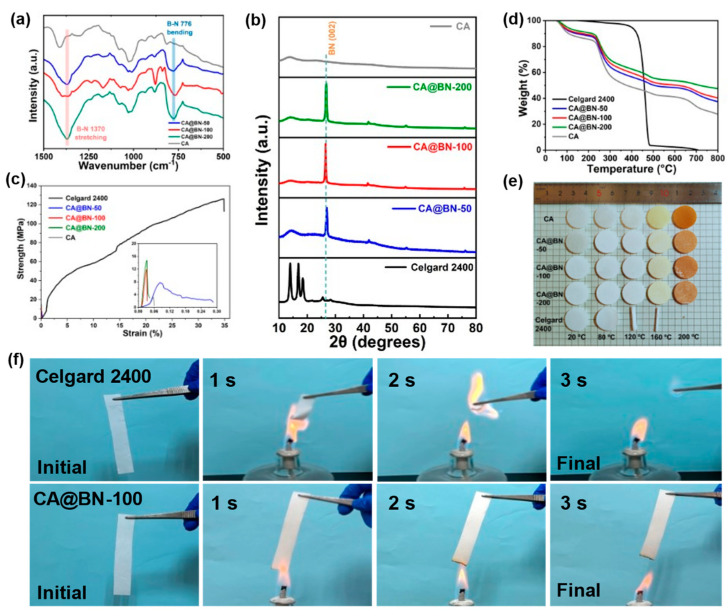
(**a**) FTIR for CA@BN-50, CA@BN-100, CA@BN-200, and CA separators. (**b**) X-ray diffraction pattern of Celgard 2400, CA@BN-50, CA@BN-100, CA@BN-200, and CA separators. (**c**) Stress-strain curve of Celgard 2400, CA@BN-50, CA@BN-100, CA@BN-200, and CA separators. (**d**) TG curves of CA, CA@BN-50, CA@BN-100, CA@BN-200, and Celgard 2400 separators. (**e**) Digital photographs of CA, CA@BN-50, CA@BN-100, CA@BN-200, and Celgard 2400 separators after heating at different temperatures. (**f**) Digital photographs of the ignition test of Celgard 2400 and CA@BN-100 separators.

The chemical compositions of samples were analyzed using FTIR, and the results are shown in Figure 2a. The CA@BN-50, CA@BN-100, and CA@BN-200 separators had two extra absorption peaks at 776 cm^−1^ and 1370 cm^−1^, respectively, compared to the CA separator. These peaks were associated with the deformation and stretching of the B-N bond [17]. It indicated that the BN was successfully coated on the CA separator.

Further, we characterized the structural composition of the samples using XRD. As shown in Figure 2b, each separator shows a distinct diffraction peak at 27.2°, attributed to the (002) crystalline surface of the BN [18,19]. In addition, no additional diffraction peaks were detected for any of the three separators, indicating that the binding of the BN powder to the CA separator occurs through physical means.

Ensuring battery safety relies heavily on the mechanical integrity of a separator. The separator must possess strong mechanical stability to withstand prolonged immersion in the electrolyte and resist external forces that potentially harm the battery’s structural integrity [20,21]. To understand the mechanical properties of the separators, we performed tensile strength tests on the samples, and the results are shown in Figure 2c. The tensile strength of the CA separator with a larger fiber aperture was only 3.1 MPa. In contrast, BN considerably improved the tensile strength of the composite separator, with the CA@BN-200 separator showing a maximum tensile strength of 14.7 MPa. At the same time, the increase in tensile strength was proportional to the thickness of the BN layer, but it also led to a decrease in elongation at break, attributed to the intrinsic stiffness of the BN material and its composite structure with fibers [22].

We further evaluated the thermal stability of the membranes using TGA. As shown in Figure 2d, the commercial Celgard 2400 separator underwent rapid decomposition at 350–500 °C with a weight loss of 97.4%. The thermal decomposition of the CA separator was divided into four main stages: the dissipation of crystalline water from the fibers (50–190 °C), decarboxylation and glycosidic bond cleavage (190–420 °C), char layer and CaCO_3_ formation (420–550 °C), and CaCO_3_ decomposition to CaO (550–800 °C) [23,24], with a residue of 28.1%. The thermal decomposition process in the coated separator was essentially the same as in the CA separator, with simultaneous PVDF decomposition at 420–550 °C [25] and no additional decomposition stages. Meanwhile, due to BNs extremely high decomposition temperature [26], the separator residue rises significantly with increasing coating thickness.

A battery separator’s thermal stability is a crucial safety component since it reduces the possibility of catastrophic battery failure, especially during extended cycles of charging and discharging [27]. A visual evaluation of the morphological changes of the separators at high temperatures was carried out to assess this attribute. To be more precise, for 30 min, CA, CA@BN-50, CA@BN-100, CA@BN-200, and Celgard 2400 were exposed to different temperatures. Digital photography taken after exposure was used to record the separators’ morphological integrity (Figure 2e). The degree of dimensional change shown in the pictures indicated the thermal stability of each separator. The CA separators CA@BN-50, CA@BN-100, and CA@BN-200 have remarkable dimensional stability up to 200 °C, significantly surpassing the Celgard 2400 separator, according to the thermal stability investigation. The remarkable heat resistance of the CA fiber that served as the foundation for these separators was highlighted by this discovery. By comparison, at lower temperatures, the commercial Celgard 2400 separator showed evidence of thermal deterioration. SEM was performed at elevated temperatures on the CA, CA@BN-100, and Celgard 2400 separators to gain a deeper understanding of the separators’ thermal performance, as shown in Figure S2. The Celgard 2400 separator’s structural integrity was compromised, and its pores were blocked at 160 °C. By contrast, even at higher temperatures, the CA and CA@BN-100 separators were distinguished by their interwoven fiber construction and retained their overall shape and pore structure. The improved safety profile of the redesigned separators in battery applications was demonstrated by their increased thermal stability. The electrochemical performance of the battery was maintained by the CA@BN separators, which help avoid thermal runaway by preserving the fiber structure and pore accessibility at high temperatures. To validate the improved thermal stability provided by the BN coating, we evaluated the heat dissipation performance of the CA and CA@BN-100 separators using an infrared thermal imager [28]. The separators were placed in an oven and heated to a constant temperature of 60 °C for two hours, as shown in Figure S3. The temperature was then recorded every eight seconds. The findings showed that the peak temperature of the CA@BN-100 separator decreased significantly from 48.3 °C to 29.1 °C, while the peak temperature of the CA separator decreased from 48.1 °C to 35.3 °C. The better heat dissipation properties of the CA@BN-100 separator are evidenced by the continuously reduced temperature gradients obtained for it. These facts show that adding BN powder to the CA separator increases thermal stability, hastens heat drainage, and lowers the possibility of thermally generated explosions within the battery. High thermal conductivity and effective heat transmission are two of BNs natural qualities [29], which are responsible for this improvement in thermal management. As such, the BN-coated CA separator becomes a viable option for applications needing high levels of safety and thermal stability.

Thermal runaway behavior is also a significant safety concern for LIBs, and separators with excellent flame-retardant properties effectively mitigate these drawbacks [30]. We conducted ignition tests on strips of commercial Celgard 2400 and modified CA@BN-100 separators of the same length to evaluate the flame retardancy of the commercial and modified separators. Digital photographs and residues during the ignition process are shown in Figure 2f. It can be observed that the commercial Celgard 2400 separator exhibited a highly flammable state when ignited, and melting droplets were observed in the residues after combustion. In contrast, the modified separator demonstrated better flame retardancy upon ignition and exhibited self-extinguishing behavior. Compared to the residues of the commercial Celgard 2400 separator, the modified separator maintained a more intact morphological structure [31].

An important feature of battery separators is the wettability of the electrolytes since this affects the separator’s ability to take up electrolytes, which is necessary for effective ion transport in LIBs [32]. An electrolyte contact angle test was conducted to evaluate this characteristic quantitatively. The CA@BN-100 separator was compared to the commercially available Celgard 2400 separator (Figure S4). The Celgard 2400 separator demonstrated strong hydrophobic properties with contact angles of 87.65°, 87.59°, and 87.09° within a 3 s capture period. The main cause of this hydrophobicity was the separator’s low surface energy, characteristic of most commercial polyolefin materials [33]. However, the CA@BN-100 separator showed notably reduced contact angles of 40.06°, 48.62°, and 51.90°, respectively. The CA@BN-100 separator’s increased wettability, which improves electrolyte retention and, consequently, promotes efficient ion transport, is demonstrated by its lower contact angle. The CA@BN-100 separator’s enhanced wettability points to a better electrolyte absorption and storage mechanism, which supports LIBs’ high-performance operation.

### 2.2. Electrochemical Performance

During the charging and discharging cycle, the separator dipped in the LIB electrolyte must sustain high voltage and retain enough electrochemical stability [34]. For this reason, we tested the electrochemical window of the three separators using linear sweep voltammetry after assembling SS||Li batteries. The scanning rate was 1 mV s^−1^, and the voltage test range was 2.0–5.5 V. In general, the first potential dramatic increase is the onset of the oxidation limit of electrochemical stability [35]. The electrolyte oxidation decomposition voltage for the SS|Celgard 2400|Li cell began at 4.3 V, according to Figure S5’s LSV curves, while modified separators showed an anodic current threshold at 4.5 V. Interestingly, the breakdown beginning of the SS|CA@BN-100|Li cell occurred at 4.7 V. These results were caused by the modified separators’ increased wettability, which increased the capacity for electrolyte retention. Better anodic stability of the improved separator made it possible to assemble it in LIBs in situations of overcharge/discharge or higher cutoff voltage.

**Figure 3:**
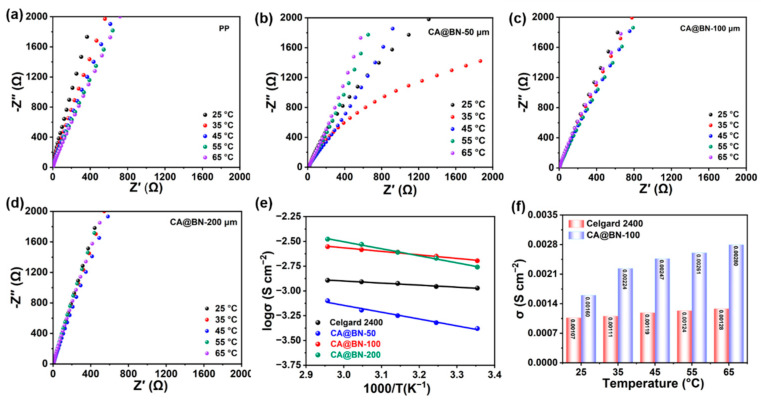
Nyquist plots for SS|separator|SS cells for (**a**) Celgard 2400, (**b**) CA@BN-50, (**c**) CA@BN-100, and (**d**) CA@BN-200 separators between 25 and 65 °C. (**e**,**f**) Ionic conductivities of the SS|separator|SS cells range from 25 to 65 °C.

Ionic migration number and ionic conductivity are crucial metrics for assessing the separator’s ability to move ions [36]. We fabricated SS||SS cells and performed impedance tests, and the results were shown in the Nyquist curves in Figure 3a–d. The bulk resistance of the Celgard 2400, CA@BN-50, CA@BN-100, and CA@BN-200 showed a steady decrease as the temperature rose from 25 °C to 65 °C.

We determined the ionic conductivities of the separators at different temperatures by fitting the AC impedance data and using Equation (1); the linear temperature-conductivity connection is shown in Figure 3e–f. When ionic conductivity was compared between the CA@BN-100 separator and the Celgard 2400, the SS|CA@BN-100|SS cell performed better, peaking at 2.80 mS cm^−1^ at 65 °C, while the SS|Celgard 2400|SS cell only managed 1.28 mS cm^−1^. The polar groups on CA fibers improve electrolyte wettability, and the BN addition refines the separator’s surface structure, optimizing the ionic transport channel. These are the main causes of the increased conductivity for the CA@BN-100.

**Figure 4:**
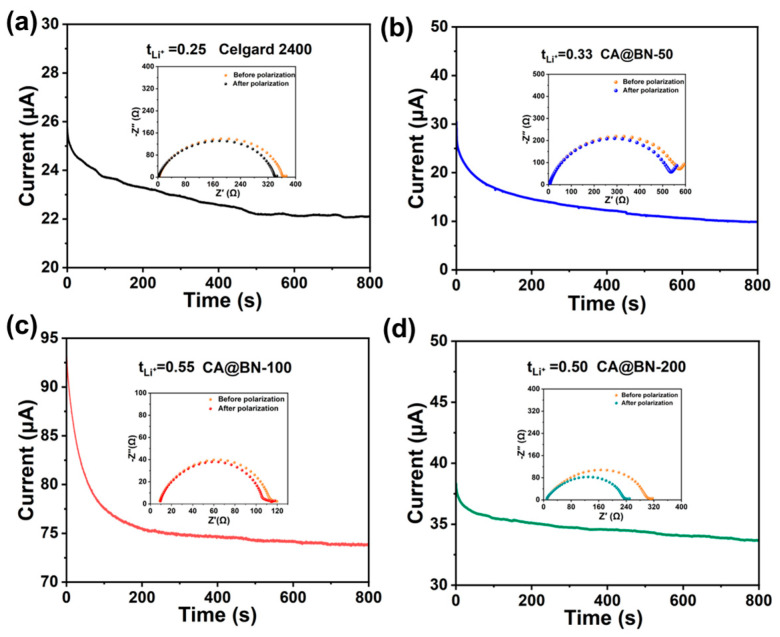
Chronoamperometry of (a) Li| Celgard 2400|Li, (**b**) Li|CA@BN-50|Li, (**c**) Li|CA@BN-100|Li, (**d**) Li|CA@BN-200|Li cells under a voltage of 10 mV (inset is the corresponding EIS profiles for the same cell before and after polarization).

One crucial metric for assessing the concentration polarization phenomena is the lithium-ion migration number. Higher t_Li_^+^ represents a better inhibition of lithium dendrite growth [36,37,38]. To evaluate the EIS curve in conjunction with chronoamperometry, we constructed Li||Li symmetrical batteries and determined the values using Equation (4). The modified separators performed better than the commercial separator, with a maximum ionic migration number (t_Li_^+^) of 0.25, as shown in Figure 4. In particular, the CA@BN-100 performed better than the Celgard 2400. After that, the CA@BN-200 had a t_Li_^+^ of 0.50. The greater electrolyte retention of the redesigned separators was responsible for the superior migration numbers, which improved cycle stability and battery longevity [39,40]. Because of the inherent mechanical limitations of pure CA fibers, the thinner BN coating, the CA@BN-50, with a t_Li_^+^ of 0.33, shows a lower migration number [41].

**Figure 5:**
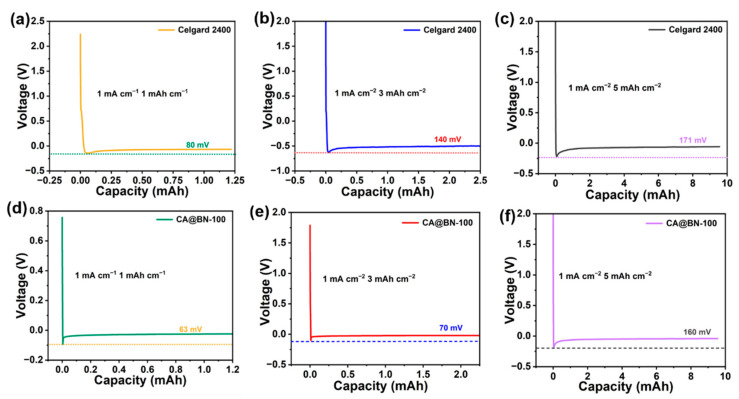
Electrochemical performance of Cu||Li half-cells with Celgard 2400 and CA@BN-100 separators in ester-based electrolyte. Voltage profile of (**a**–**c**) Cu|Celgard 2400|Li and (**d**–**f**) Cu|CA@BN-100|Li cells at 1 mA cm^−2^. The area capacity is 1 mAh cm^−2^, 3 mAh cm^−2^, and 5 mAh cm^−2^.

Using CA@BN-100 and Celgard 2400 separators, we assembled Cu|separator|Li half-cells and investigated lithium nucleation on copper at current densities of 1 mA cm^−2^ with area capacities of 1, 3, and 5 mAh cm^−2^, as shown in Figure 5. The difference between the lowest instantaneous potential and the stable cycle potential, representing the level of metallic lithium deposition on the copper foil, is known as the nucleation overpotential value [42]. One of the main causes of the creation of lithium dendrites is a kinetically unfavorable process on the copper foil caused by a greater overpotential difference, resulting in a larger nucleation barrier for metallic lithium [19]. A reduced overpotential suggests that the copper foil has a uniform layer of lithium metal deposited, which is good for the battery’s cycle stability. The voltage curve of the Cu|separator|Li half-cells in Figure 5 exhibited a steep decline associated with the metallic lithium nucleation process. Then, the voltage progressively increased and stabilized, depending on the electrochemical polarization, ion-solvent dissociation, and stable cycle potential of mass transfer. The Cu|Celgard 2400|Li battery’s nucleation overpotential values under the same current density and varied area capacities were 100 mV, 140 mV, and 171 mV, respectively, as seen in Figure 5a–c. The Cu|CA@BN-100|Li battery had a similar voltage difference of 40 mV, 70 mV, and 160 mV under the same conditions (Figure 5d,e), which was significantly less than the Cu|Celgard 2400|Li batteries. It suggested the improved separator supports the battery’s lithium anode interface stability.

Using constant current cycling tests on Cu|Celgard 2400|Li and Cu|CA@BN-100|Li batteries at 1 mA cm^−2^ and a deposition capacity of 1 mAh cm^−2^, we examined the separator’s impact on lithium plating/stripping. As illustrated in Figure S6, the Cu|CA@BN-100|Li battery demonstrated strong cycle stability after 100 cycles, maintaining a Coulombic efficiency (CE) of around 98%, but the Cu|Celgard 2400|Li battery experienced a decline to roughly 74%. It was due to the Cu|CA@BN-100|Li battery having a lower nucleation barrier, which influenced the nucleation of lithium and raised the battery’s CE.

The battery’s CE is also impacted by the surface shape of the lithium deposition. So, we used an ester-based electrolyte to study the lithium metal morphology on copper foil at nucleation current densities of 1 mA cm^−2^ and capacities of 1, 3, and 5 mAh cm^−2^, as shown in Figure S7. Under the same starting conditions, uniform lithium particles were seen on the copper foil with the modified separators, eventually developing into a smooth surface at 5 mAh cm^−2^. Conversely, at the identical deposition conditions, the Celgard 2400 separator displayed dendritic lithium structures, which raised the possibility of damage to the separator and LIB failure. This was attributed to the excellent physical stability of the BN-coated separator, which reduced the formation of dendritic structures, thereby maintaining electrochemical performance, reducing the risk of short circuits, and extending the battery’s cycle life.

One significant way to confirm the stability of the battery interface is to test lithium symmetrical batteries for charge and discharge at constant current. The Li||Li symmetrical batteries with Celgard 2400 and CA@BN-100 separators underwent a long-term constant current charge and discharge test with a current density of 0.5 mA cm^−2^, as illustrated in Figure S8. After 1200 h of cycling, the battery constructed with the BN-coated separator demonstrated good electrochemical performance with a stable voltage platform and cycling process. The Celgard 2400 battery exhibited the lowest polarization at 0.05 V within the first 200 h of operation. There were also notable voltage fluctuations and a short circuit around 680 h. On the other hand, after 900 h, the battery that was manufactured using the improved separator was able to maintain a steady voltage of 0.03 V. According to SEM examinations of the lithium-deposited copper foil, it suggested that the CA@BN-100 separator stabilized the battery’s electrolyte-electrode interface, with small voltage fluctuations indicating efficient dendrite suppression during lithium plating/stripping.

The above results show that the unique structure of BN in the modified separator can effectively inhibit the growth of lithium dendrites. It was due to the high hardness of BN, which reduces the physical damage of the CA separator during the cycling process.

**Figure 6:**
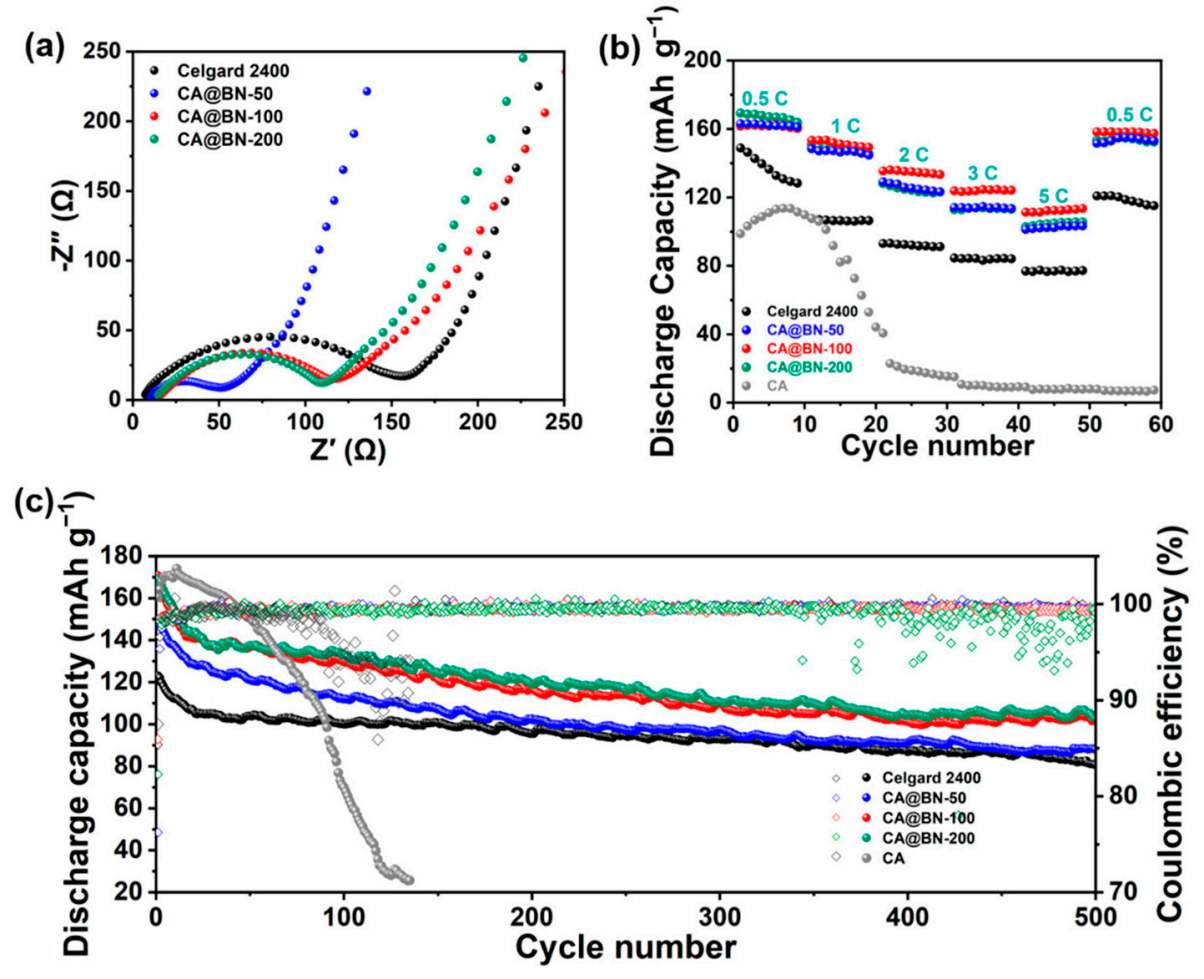
(**a**) EIS of LFP||Li cells with Celgard 2400, CA@BN-50, CA@BN-100, and CA@BN-200 separators. (**b**) Rate capability of LFP||Li cells. (**c**) Long-term cycling performances of LFP||Li cells at 1 C.

We put up a complete battery utilizing a Li anode and an LFP cathode in order to investigate the CA@BN-100 separator’s potential in real applications. Initially, we assessed the electrolyte’s compatibility with the separator using the EIS curve test [43]. The entire batteries with changed separators (CA@BN-50, CA@BN-100, and CA@BN-200) were depicted in Figure 6a. These batteries had lower interface impedances and semicircle diameters, with specific resistance values of 40 Ω, 100 Ω, and 94 Ω, respectively. These values are mostly related to the coating thickness. By comparison, the battery that was constructed using the Celgard 2400 separator had an interface resistance of 148 Ω. This outcome was explained by the improved separator’s superior electrolyte wettability, which enhanced the electrolyte and separator interface compatibility.

The rate performance of complete batteries with three coated separators within 2.5–4 V, CA, and Celgard 2400 is shown in Figure 6b. Because higher concentration polarization led to overpotential and increased Ohmic polarization, the discharge capacity fell as current density increased [44]. One of them, the battery constructed with the CA separator, proved problematic in LIBs since it broke down after just 20 cycles and failed to charge and discharge at varying current densities. Second, at current densities of 0.5 C, 1 C, 2 C, 5 C, and 0.5 C, respectively, the LFP complete battery made with the Celgard 2400 separator similarly displayed the maximum values of 150 mAh g^−1^, 107 mAh g^−1^, 93 mAh g^−1^, 84 mAh g^−1^, 75 mAh g^−1^, and 121 mAh g^−1^. Because of strong internal polarization, the battery constructed with the Celgard 2400 separator rapidly lost the capacity to 75 mAh g^−1^ at a current density of 5 C. The full batteries with changed separators (CA@BN-50, CA@BN-100, and CA@BN-200), on the other hand, maintained capacities of 103 mAh g^−1^, 112 mAh g^−1^, and 105 mAh g^−1^ at a current density of 5 C, with a capacity retention rate of 64%, 70%, and 63% in comparison to 0.5 C, significantly exceeding the 50% capacity retention rate of the Celgard 2400 separator. This was achieved by using the three different separators, CA@BN-50, CA@BN-100, and CA@BN-200. The improved ionic conductivity and good interface compatibility of the redesigned separators are the primary causes. Figure S9 indicates that the battery with the CA@BN-100 separator exhibited an excellent discharge platform and higher discharge voltage across all tested rates, reflecting its outstanding rate capability and energy density.

Stable functioning during charging and discharging plays a direct role in increasing battery life. As a result, we used three modified separators, CA, and entire batteries made using LFP as the cathode, and we ran long-term charge and discharge cycle tests on them with a voltage range of 2.5–4 V and a current density of 1 C. The battery built using the CA separator had a capacity degradation to 0 mAh g^−1^ within 150 cycles, as seen in Figure 6c. Within an 800-circle cycle, the batteries built with the improved separators following BN coating continued to exhibit a high discharge capacity. After 800 cycles, the battery’s Coulombic efficiency curve diverged due to the commercial Celgard 2400 separator, resulting in a discharge capacity decay of 83 mAh g^−1^. Conversely, the modified separator demonstrated the highest capacity retention rate, with a discharge capacity of 89 mAh g^−1^ after cycling CA@BN-50, CA@BN-100, CA@BN-200, and Celgard 2400 separators at varying C-rates.

LFP||Li batteries with Celgard 2400 and CA@BN-100 were evaluated for cyclic stability at 60 °C in order to verify the thermal stability of the separators. The ionic conductivity of the separator increases as the temperature rises to 60 °C, and both batteries’ initial discharge capacity reached 170 mAh g^−1^ at a current density of 1 C. There is a progressive decline in the discharge capacity as the number of cycles increases. The battery paired with the Celgard 2400 separator displayed a significant variation in its Coulombic efficiency. With an average Coulombic efficiency of 97%, the improved separator’s capacity retention rate reached 72.3% after 1000 cycles. On the other hand, the Celgard 2400 separator’s capacity retention rate was a mere 41%. These numbers show that at greater temperatures, the battery fitted with the improved separator can continue to have better cycle stability.

## 3. Conclusions

To summarize, our study focused on a coated separator that incorporates calcium alginate fiber and BN, resulting in enhanced safety and cycle performance of lithium-ion batteries. The preparation process of the coated separator is straightforward and ecologically friendly, in contrast to other separator modification procedures. It does not require time-consuming experimental treatment phases. Additionally, calcium alginate fiber is derived from the ocean and possesses exceptional flame-retardant characteristics. Furthermore, BN powder possesses adequate mechanical strength to inhibit the proliferation of lithium dendrites, hence improving the stability of the lithium anode interface in Li||Li cells [40,45]. This leads to the successful plating and stripping of lithium, allowing for 1000 stable cycles at a current density of 0.5 mA cm^−2^. The interwoven topology of the BN-CA fiber network provides numerous paths for ion movement, hence enhancing the retention of electrolytes, compatibility at the electrode interface, and electrochemical stability. The improved separator enhances the ionic conductivity to 2.80 mS cm^−1^ at 65 °C and achieves a lithium-ion migration number of 0.55. Additionally, the LFP||Li battery, when combined with the modified separator, successfully completed 800 stable charge and discharge cycles. Ultimately, as a result of BNs exceptional thermal conductivity, the improved separator demonstrates outstanding thermal stability. The LFP||Li battery, when used with the CA@BN-100 separator, can last more than 1000 cycles at a temperature of 60 °C and a charge/discharge rate of 1 C while retaining a Coulombic efficiency of 97% (Figure S10). In summary, this study presents novel approaches for modifying lithium-ion battery separators.

## 4. Experimental Section

### 4.1. Materials and Characterization

The CA fibers were acquired from Qingdao Yuanhai New Material Technology Co., LTD (Qingdao, China). The source of boron nitride (BN) was Shanghai Aladdin Chemical Reagents Co., LTD (Shanghai, China). The N, N-Dimethylformamide was acquired from Sinopharm Chemical Reagent Company (Shanghai, China). Carbonate electrolyte (1 M LiPF_6_ in EC: DMC: DEC = 1:1:1, *v*/*v*/*v*) and polypropylene separators (Celgard 2400) were acquired from Duo Duo Chemical Technology Co., LTD (Suzhou, China), while LiFePO_4_ (LFP), Keqin Black, PVDF, and N-methyl-2-pyrrolidone (NMP) were obtained from Hefei Kejing Material Technology Co., LTD (Hefei, China). The reagents were used in their original form without any additional purification.

The morphology of the samples was analyzed using Scanning Electron Microscopy and Energy-Dispersive X-ray Spectroscopy (SEM and EDS, Quanta 250 FEG, FEI NanoPorts, Hillsboro, ON, USA). The structure and mechanical properties of the separators were evaluated using Fourier Transform Infrared Spectroscopy (FTIR, Nicolet iS50, THERMO FISHER, Waltham, MA, USA), X-ray Diffraction (XRD, DX2700, Dandong Haoyuan Instrument Co., Ltd., Dandong, China), and a universal tensile tester (WDW-2T, Zhong Ke Instrument Co., Ltd., Jinan, China). The wettability of the separators was assessed by conducting contact angle measurements with the electrolyte. The thermal stability of separators was measured by the thermogravimetric analyzer (TG, TGA2, Mettler Toledo, Zurich, Switzerland) and thermal infrared imaging. Flammability tests were performed on the separators and compared to commercially available Celgard 2400 separators (Celgard Limited Liability Company, Charlotte, NC, USA).

### 4.2. Preparation Method

#### 4.2.1. Fabrication of CA Separator

Three to five-millimeter pieces of calcium alginate fiber were cut. Weigh out 2.25 g of these fiber pieces, and then soak them for 12 h in a beaker filled with adequate deionized water. Next, move the fiber that has been soaked to a regular fiber disintegrator and let it dissolve for ten minutes. After that, the crumbled pulp is put into a paper mold to be formed. Ultimately, a combination of vacuum hot pressing and drying yields calcium alginate paper.

#### 4.2.2. Preparation of the CA@BN Separator

Boron nitride nanopowder was mixed with polyvinylidene fluoride powder in the ratio of 4:1. When BN:PVDF = 3:1, as shown in Figure S11, excess PVDF would clog the pores and cause the separator to fail. Therefore, we adopted the work of Rodriguez et al. [46] to coat the separator with BN:PVDF = 4:1. and then N,N-dimethylformamide was added to make a homogeneous dispersion. The paste was uniformly applied to one or both sides of the rectangular paper using a coating applicator with thicknesses of 50 μm, 100 μm, and 200 μm, respectively. The laminated paper was dried in an oven at 60 °C. The dried material was cut into different segments using a cutter and labeled CA@BN-50, CA@BN-100, and CA@BN-200 for spare use.

### 4.3. Electrolyte Uptake Ratio

The quantification of electrolyte absorption is determined using the subsequent equation:

(1) $$E_{V}=\frac{\left(W_{1}-W_{0}\right)}{W_{0}}\times 100\%$$

*W*_0_ and *W*_1_ indicate the initial and final mass of the separator after a 2 h infiltration in the electrolyte, respectively.

The porosity was calculated using the following equation:

(2) $$P=\frac{\left(W_{wet}-W_{dry}\right)}{\rho V}\times 100\%$$

where *W_wet_* and *W_dry_* represent the mass of separator soaked in butanol for 2 h and the separator after drying. *ρ* and *V* are the density of butyl alcohol and the dried separator volume, respectively.

### 4.4. Electrochemical Test

The CHI760E electrochemical workstation (China) was used to perform Electrochemical Impedance Spectroscopy (EIS), Linear Sweep Voltammograms (LSV), and the Cyclic Voltammograms (CV). The Electrochemical Impedance Spectroscopy (EIS) was analyzed throughout the frequency range of 0.1 Hz to 100 kHz using an applied alternating current (AC) of 5 mV.

The LSV underwent testing using a voltage range of 3.0–5.5 V and a scan rate of 1 mV s^−1^. The separator is positioned between a cell, with the stainless steel serving as the working electrode and the lithium foil acting as the counter and reference electrode. The cyclic voltammetry (CV) experiments were performed on LFP||Li cells using Celgard 2400 and CA@BN separators. The voltage range for the testing was 2–4 V, and the scan rate was set at 0.1 mV s^−1^.

The ionic conductivity of the separator was evaluated by EIS of the assembled stainless steel (SS)||SS cells. It was calculated using the following formula:

(3) $$\sigma =\frac{L}{R_{b}\times A}$$

*σ* is the ionic conductivity, *L* is the thickness of the separator, *R_b_* is the bulk resistance of the separator, and *A* is the contact area of the *SS* electrode with the separator.

The Li^+^ transference number was measured in a Li||Li symmetric cell by combining the AC impedance and direct-current polarization (with a polarization voltage of 10 mV) and was calculated using the following formula:

(4) $$t^{+}=\frac{I_{ss}(∆V-I_{0}R_{0})}{I_{0}(∆V-I_{ss}R_{ss})}$$

*t^+^* is the transference number of lithium ions, *I*_0_ and *I_SS_* represent the initial current and steady-state current, respectively, Δ*V* is the applied polarization, and *R*_0_ and *R_SS_* represent the initial interface resistance and the steady-state resistance in the EIS, respectively.

The performance of the whole cells was evaluated by utilizing CR2016 coin cells with LFP as the cathode and Li foil as the anode. The cathode was created by drying a mixture of LFP, Keqin Black, and PVDF in a weight ratio of 8:1:1. The charge and discharge characteristics were evaluated using a LAND battery test system within a potential range of 2.5–4.0 V. The Li metal anode has a diameter of 15.6 mm, while the LFP cathode has a diameter of 13 mm. The loading of active material in the LFP cathode was 1.09 mg cm^−2^.

## Data Availability

Data are contained within the article and Supplementary Materials.

## Supplementary Materials

The following supporting information can be downloaded at: https://www.mdpi.com/article/10.3390/molecules29225311/s1, Figure S1: (a–c) Cross-sectional SEM image of CA@BN-50 separator and EDS elemental mapping of F, O, N, B. (d–f) Cross-sectional SEM image of CA@BN-100 separator and EDS elemental mapping of F, O, N, B. (g–i) Cross-sectional SEM image of CA@BN-200 separator and EDS elemental mapping of F, O, N, B.; Figure S2: SEM images of (a–c) CA, (d–f) CA@BN-100, and (g–i) Celgard 2400 separators after heating at 120 °C, 160 °C, and 200 °C; Figure S3: Thermal infrared images of CA@BN-100 and CA separators; Figure S4: Contact angles of Celgard 2400 and CA@BN-100 separators with the liquid electrolyte; Figure S5: LSV of SS|separator|Li cells; Figure S6: CE of Cu|Celgard 2400|Li and Cu|CA@BN-100|Li cells at 1.0 mA cm^^−2^^ for 1.0 mAh cm^−2^; Figure S7: SEM characterization of Li plating on the Cu substrate with (a–c) CA@BN-100 and (d–f) commercial Celgard 2400 separators at 1.0 mA cm^−2^ for 1.0 mAh cm^−2^, 3.0 mAh cm^−2^, 5.0 mAh cm^−2^; Figure S8: Voltage profiles of the Li|CA@BN-100|Li and Li|Celgard 2400|Li symmetrical cells at 0.5 mA cm^−2^, 1 mAh cm^−2^; Figure S9: the discharge voltage versus capacity curves of LFP||Li cells using CA, CA@BN-50, CA@BN-100, CA@BN-200, and Celgard 2400 separators at various rates; Figure S10: High-temperature cycling stability of LFP||Li cells with CA@BN-100 and Celgard 2400 separators at a charge/discharge rate of 1 C; Figure S11: SEM image of CA@BN separator (BN:PVDF = 3:1); Table S1: The content of element.
