# Supplementary material for: Calcium Alginate Fibers/Boron Nitride Composite Lithium-Ion Battery Separators with Excellent Thermal Stability and Cycling Performance
Source: Molecules. 2024 Nov 11;29(22):5311. doi: 10.3390/molecules29225311 (PMC11596189; doi:10.3390/molecules29225311)
Supplement: Supplementary file 1 [file molecules-29-05311-s001.zip › molecules-3279079-supplementary.pdf]

Supplementary Information

Calcium Alginate Fibers/Boron Nitride Composite Lithium-Ion Battery Separators with Excellent Thermal Stability and Cycling Performance

Xing tian[a,b*], Hailing Shi[b], Linfeng Wang[b], Lupeng Shao[a], Liwen Tan[b*]

[a]State key Laboratory of Biomaterials and Green Papermaking, Qilu University of Technology, Jinan,250306, P. R. China.

[b]State Key Laboratory of Biofibers and Eco-textiles, College of Materials Science and Engineering, Institute of Marine Bio-based Materials, Qingdao University, Qingdao 266071, P. R. China.

Corresponding author

Xing Tian-State key Laboratory of Biomaterials and Green Papermaking, Qilu University of Technology, Jinan,250306, P. R. China.*

State Key Laboratory of Biofibers and Eco-textiles, College of Materials Science and Engineering, Institute of Marine Bio-based Materials, Qingdao University, Qingdao 266071, P. R. China.

E-mail:xingtian1982@126.com.

Liwen Tan- State Key Laboratory of Biofibers and Eco-textiles, College of Materials Science and Engineering, Institute of Marine Bio-based Materials, Qingdao University, Qingdao 266071, P. R. China.*

E-mail:liwent@qdu.edu.cn.

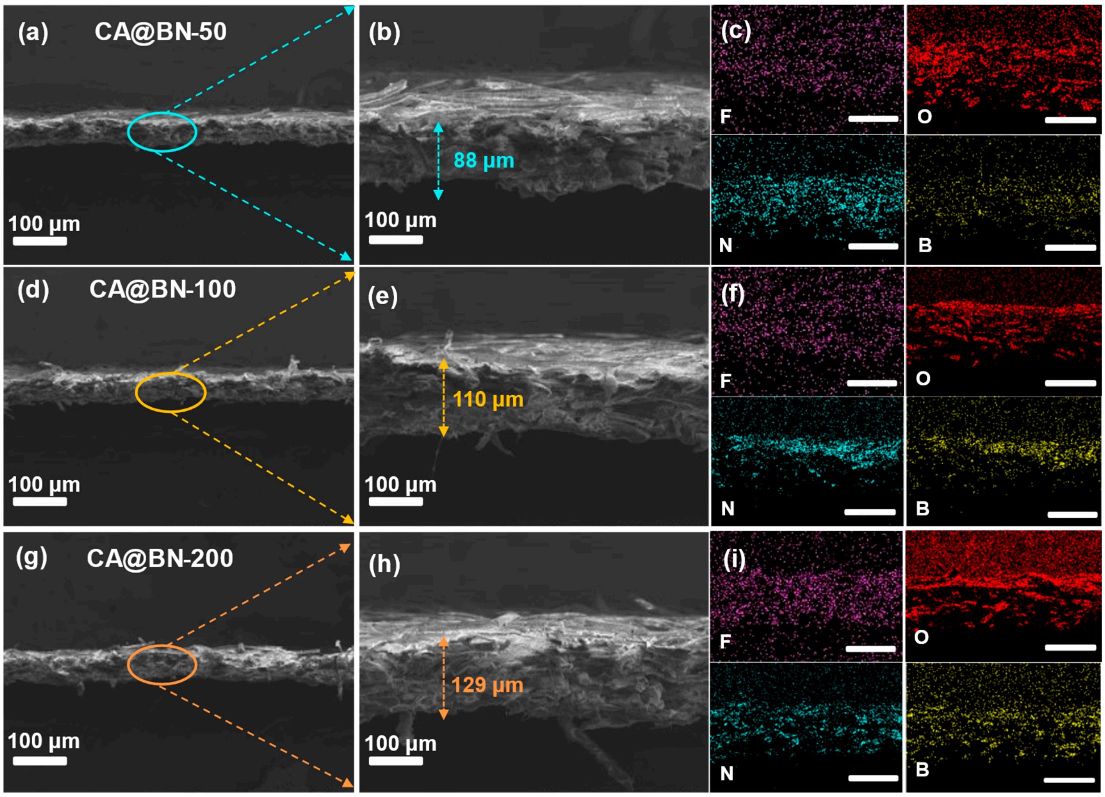

Figure S1. (a-c) Cross-sectional SEM image of CA@BN-50 separator and EDS elemental mapping of F, O, N, B. (d-f) Cross-sectional SEM image of CA@BN-100 separator and EDS elemental mapping of F, O, N, B. (g-i) Cross-sectional SEM image of CA@BN-200 separator and EDS elemental mapping of F, O, N, B.

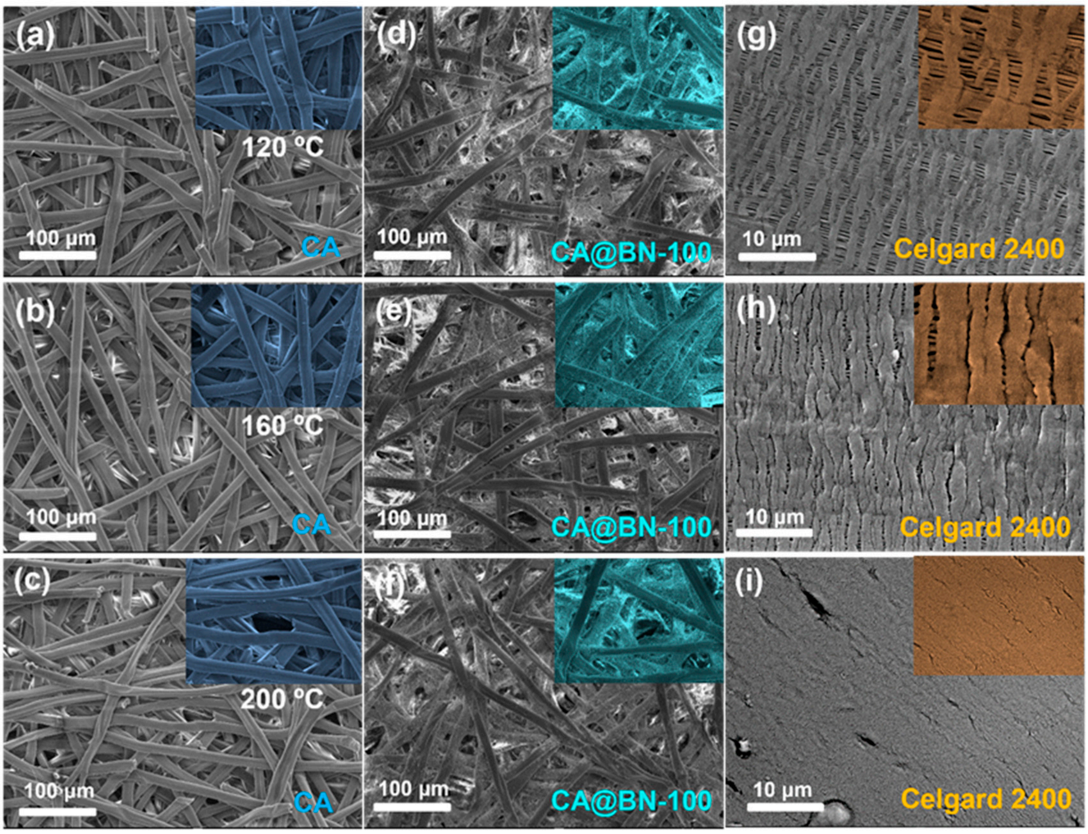

Figure S2. SEM images of (a-c) CA, (d-f) CA@BN-100, and (g-i) Celgard 2400 separators after heating at 120 ºC, 160 ºC, and 200 ºC.

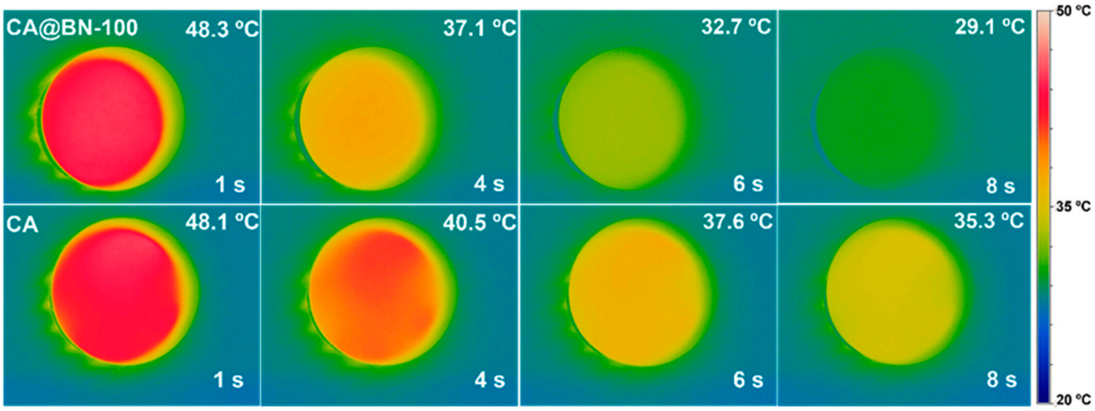

Figure S3. Thermal infrared images of CA@BN-100 and CA separators.

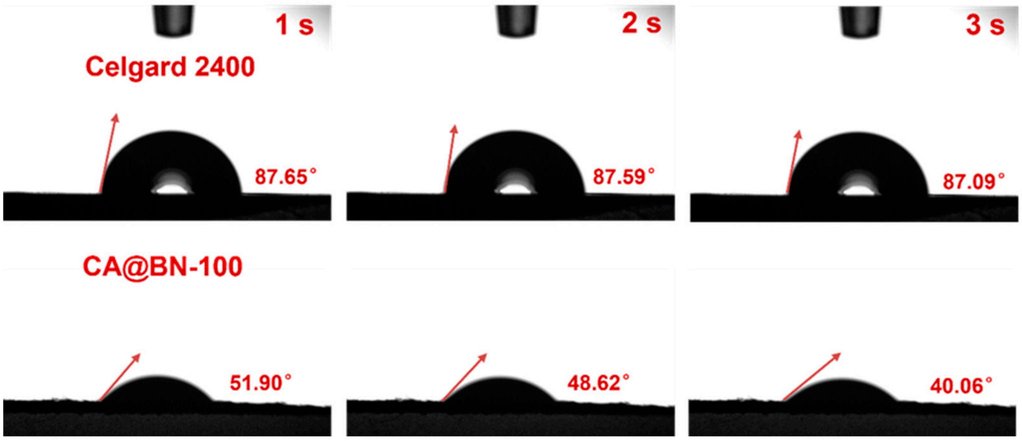

Figure S4. Contact angles of Celgard 2400 and CA@BN-100 separators with the liquid electrolyte.

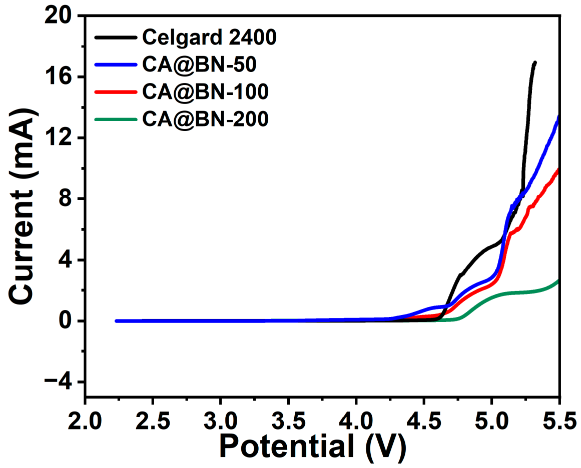

Figure S5. LSV of SS|separator|Li cells.

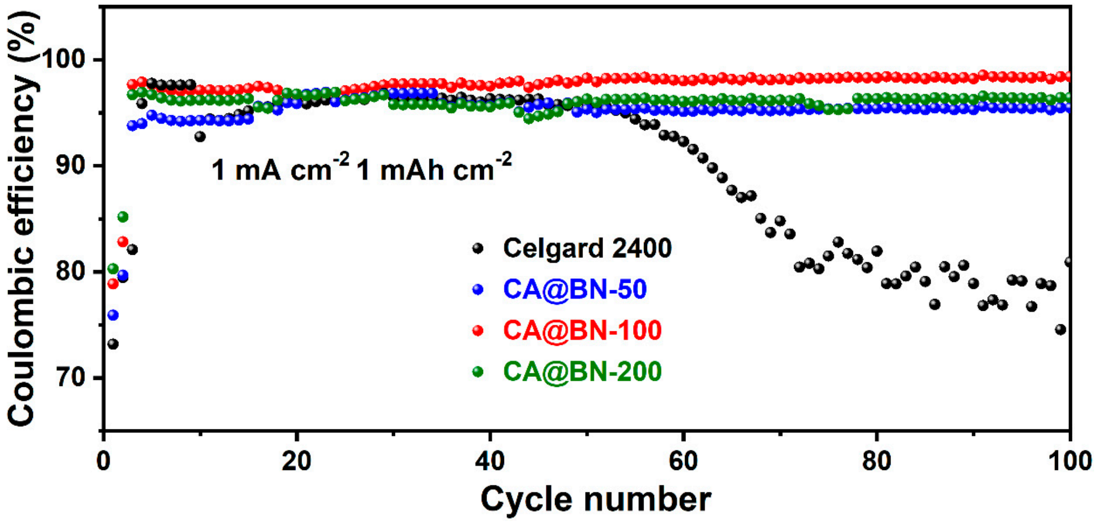

Figure S6. CE of Cu|Celgard 2400|Li and Cu|CA@BN-100|Li cells at 1.0 mA cm^{-2} for 1.0 mAh cm^{-2}.

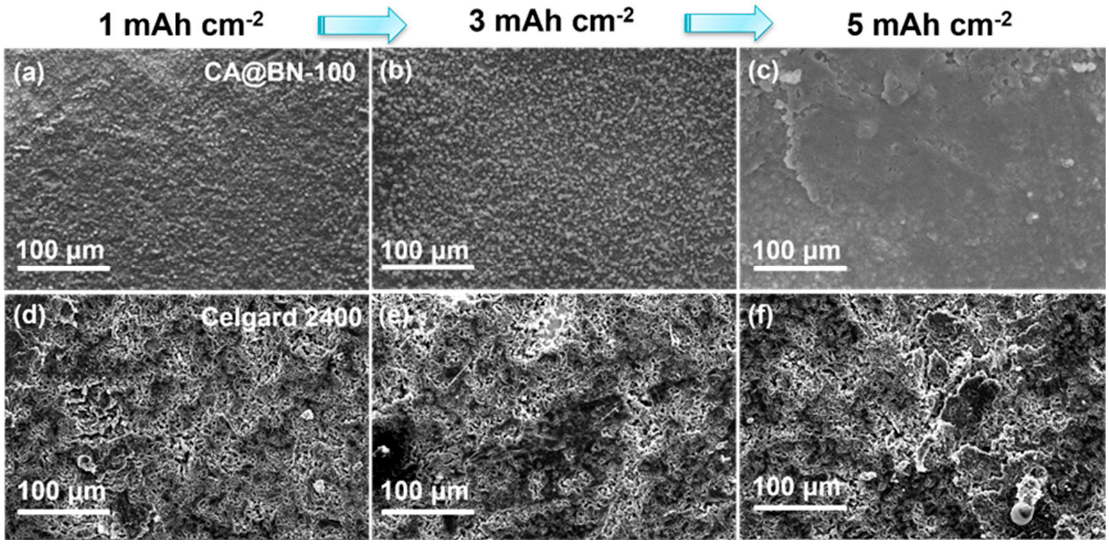

Figure S7. SEM characterization of Li plating on the Cu substrate with (a-c) CA@BN-100 and (d-f) commercial Celgard 2400 separators at 1.0 mA cm^{-2} for 1.0 mAh cm^{-2}, 3.0 mAh cm^{-2}, 5.0 mAh cm^{-2}.

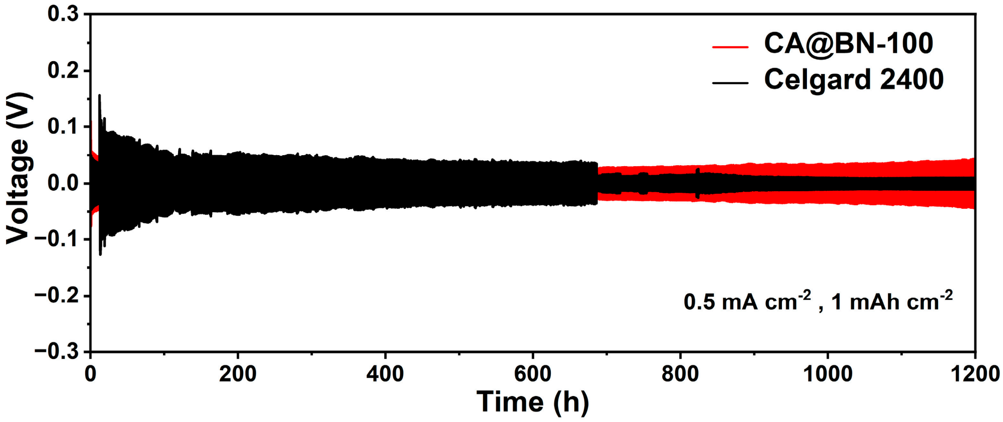

Figure S8. Voltage profiles of the Li|CA@BN-100|Li and Li|Celgard 2400|Li symmetrical cells at 0.5 mA cm^{-2}, 1 mAh cm^{-2}.

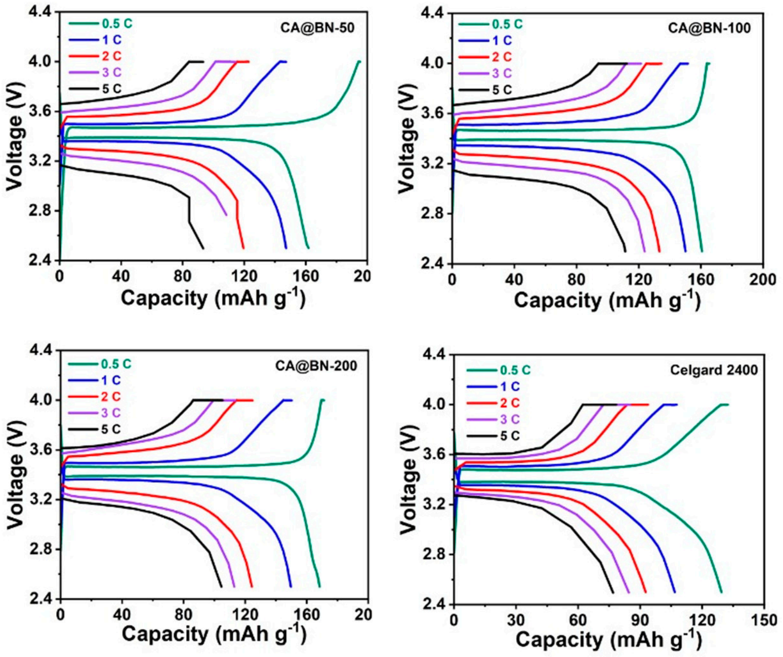

Figure S9. The discharge voltage versus capacity curves of LFP||Li cells using CA, CA@BN-50, CA@BN-100, CA@BN-200, and Celgard 2400 separators at various rates.

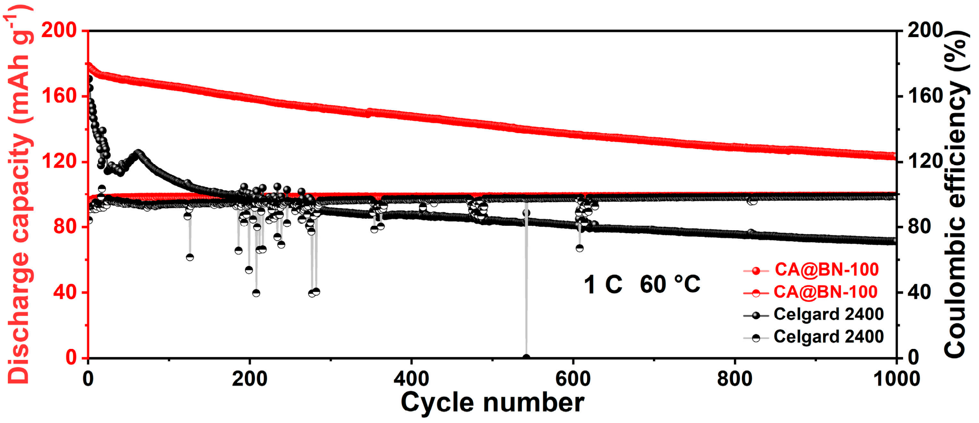

Figure S10. High-temperature cycling stability of LFP||Li cells with CA@BN-100 and Celgard 2400 separators at a charge/discharge rate of 1 C.

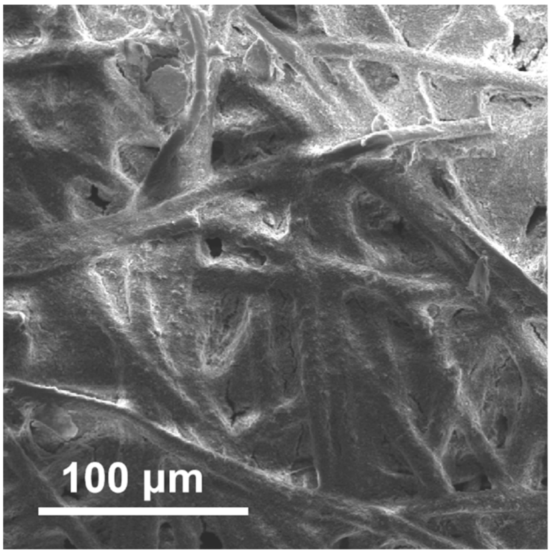

Figure S11. SEM image of CA@BN separator (BN: PVDF = 3:1).

Table S1. The content of element

Samples	B	C	N	O	Ca	F
CA@BN-100 surface	32.07	12.9	45.89	8.07	0.64	0.43
CA@BN-50 cross-section	18.72	32.22	23.35	22.54	1.98	1.19
CA@BN-100 cross-section	17.35	37.27	18.91	22.34	3.1	1.03
CA@BN-200 cross-section	14.85	37.24	13.14	29.57	2.91	2.29
